# Patient activation levels and socioeconomic factors among the Amazonas population with diabetes: a cross-sectional study

**DOI:** 10.1186/s12913-023-10529-0

**Published:** 2024-02-06

**Authors:** Elisa Brosina de Leon, Hércules Lázaro Morais Campos, Natália Barbeiro Santos, Fabiana Almeida Brito, Fabio Araújo Almeida

**Affiliations:** 1 Program in Human Movement Sciences, Faculty of Physical Education and Physiotherapy, Federal University of Amazonas, Manaus, Brazil; 2 Institute of Health and Biotechnology, Federal University of Amazonas, Coari, Brazil; 3 Department of Health Promotion College of Public Health University, Nebraska Medical Center, Omaha, USA

**Keywords:** Diabetes Mellitus Type 2, Patient Participation, Primary Health Care, Public Health

## Abstract

**Background:**

The presence of chronic conditions such as type 2 diabetes mellitus (T2DM) requires behavioral lifestyle changes mediated by individuals’ motivation for change and adherence to treatment. This study aims to explore activation levels in individuals with T2DM treated in primary care facilities and to identify the association between demographic, clinical, psychosocial factors, and patient activation amongst populations in the Brazilian state of Amazonas.

**Methods:**

SAPPA is a cross-sectional study conducted in Amazonas, approved by the Universidade Federal do Amazona’s IRB in Brazil. Individuals with T2DM were evaluated in their homes (n = 4,318,325). The variables were sex, age, skin color, education level; health-related variables such as body mass index, nutritional behavior, and frequency of physical activity. Measures related to patient self-management behaviors over the past 6 months (Patient Activation Measure – PAM-13) were included in the survey. Descriptive and frequency data are presented as mean (standard deviation (SD)) or numeric percentage). Statistical testing was performed using IBM SPSS V.26, and a *p*-value of < 0.050 showed significance. Activation levels were dichotomized into low activation (Levels 1 and 2) and high activation (Levels 3 and 4). A multivariate linear model assessed the association between the PAM-13 score and the following variables: age, sex, BMI, skin color, number of comorbidities, burden of symptoms, and number of medications.

**Results:**

Logistic regression analyses indicated a statistically significant association between sex, age, education, self-rated health, and general satisfaction with life. men were 43% more likely to score lower levels (*p* < 0.001). The results also indicated that advanced age had lower PAM levels (*p* < 0.001). Participants with fewer years of education were 44% more likely to have lower levels of PAM (*p* = 0.03). Worse self-rated health (*p* < 0.001) and lower general life satisfaction (*p* = 0.014) were associated with lower PAM levels.

**Conclusions:**

Low patient activation was associated with worse sociodemographic, health, and psychological conditions in the Amazon population. The low level of patient activation observed in this sample highlights an important impediment to diabetes disease management/self-management in disadvantaged populations.

**Supplementary Information:**

The online version contains supplementary material available at 10.1186/s12913-023-10529-0.

The International Diabetes Federation estimated that 537 million adults live with Diabetes Mellitus (DM), including Type 2 DM (T2DM), the most common type, accounting for over 90% of all cases worldwide [1]. As one of the most prevalent non-chronic diseases, T2DM affects 6% of the adult population [2], and it has also been observed as making a progressive increase in Brazilian adults over time [3]. In 2015, there were 14.3 T2DM cases., with a projected increase to 23.3 million cases in 2040 [4].

The causes of type 2 diabetes are not entirely understood, but there is a strong link between overweight and obesity, increasing age, skin color, and family history. Contributors to type 2 diabetes risk include polygenic and environmental triggers [1]. Diabetes care demands changing the role perspective of health care systems, health care professionals, and T2DM patients to improve diabetes-related outcomes [5]. The cornerstone of T2DM management is promoting a lifestyle that includes a healthy diet, regular physical activity, smoking cessation, and maintenance of healthy body weight [1, 6]. This approach requires continuous, proactive, and integrated responses and actions from healthcare systems and professionals to stabilize health conditions through effective and efficient quality control [7].

In addition, in patient-centered healthcare systems, patients are expected to engage in their care, which requires sufficient knowledge, motivation, skills, and confidence to properly manage their disease– in other words, they need a sufficient level of “activation” [8]. Patient activation refers to one’s internal readiness and capabilities to undertake health-promoting actions [9]. Higher patient activation levels significantly improve T2DM self-management [10]. However, different factors affect patient activation, highlighting the importance of individual characteristics. Patient activation can be affected by employment status [11], age, health status [11–13], and education level [14]. Also, self-management highlights the relevance of psychological factors, positing that changes in health behavior require motivational and self-regulatory skills [15]. Despite this evidence, a deep understanding of defining and summarizing determinants of patient activation is still lacking.

Very little is known about which determinants– whether demographic, psychosocial, or environmental– are relatively more important when it comes to predicting patient activation [15], particularly, in low-income populations. Income, education, and occupation show a graded association with diabetes prevalence and complications across all levels of socioeconomic factors [16].

Cities in the Brazilian Amazon are unique in several ways when compared to other cities in Brazil. They have a lower degree of development and great economic and social fragility due to the scarcity of income-generating agents and migration. This situation worsens when moving away from the urban areas toward rural communities located on the banks of rivers and/or roads and surrounding areas. In these places, access to goods and services becomes very difficult, increasing social risks [17].

All these inequalities could affect activation, quality of care, and the life of patients. This study hypothesizes that vulnerable people, including low education level and low income, will present low activation. Based on the knowledge that individuals with type 2 diabetes mellitus require a sufficient level of “activation” to truly engage in treatment [8], this study aims to explore activation levels in individuals with T2DM treated in primary care facilities and to identify the association between demographic, clinical, psychosocial factors and patient activation amongst populations in the Brazilian state of Amazonas. Our findings can inform decision-makers and health practitioners about targetting patient activation interventions for such populations [11].

## Methods

### Setting

This is a cross-sectional study carried out with populations residing in Amazonas, Brazil. The data used in the current study were collected during the development of the Study of Health in Primary Care of the Amazon Population (SAPPA), conducted in Amazonas, between August 2020 and April 2021 [18]. The ethical approval (registration: 4.318.325 and 4.994.196) was granted by the Research Ethics Committee with humans (IRB) of the Amazonas Federal University, and the study followed the bioethics principles according to the 510/16 resolution of the National Health Council. All participants signed consent forms.

**Fig. 1:**
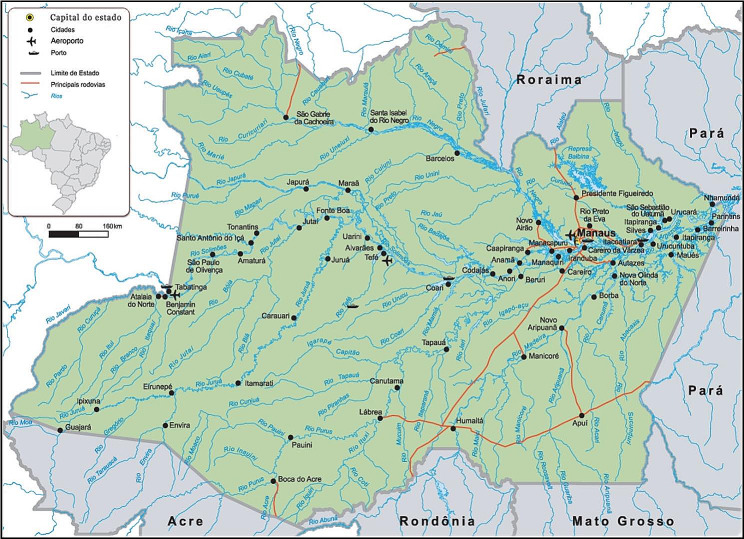
Amazonas map

SAPPA is the first large-scale study of the Amazon population focused on Chronic Diseases Care. SAPPA is carried out at Primary Care Health Units located in urban and rural areas in every city in the state of Amazonas. The first data collection included eight cities in the Metropolitan Region of Manaus and the second included four cities located in the Middle Solimões (Fig. 1). The project SAPPA was designed to provide data on the health care model offered to patients diagnosed with T2DM who are treated at Primary Care Health Units, and how this care has impacted their health and behaviors. The aim of the project is not only to build an understanding of the real situation but also to provide scientific data to support and study policy changes that may affect and improve patient care.

The current study was conducted in Primary Care Health Units in eight Amazonas cities: Iranduba, Itacoatiara, Manacapuru, Novo Ayrão, Presidente Figueiredo, Rio Preto da Eva, Silves, Itapiranga, and Coari. Primary Care Health Units selection were drawn according to the National Register of Health Establishments.

Community Health Workers provided a list of potential participants based on their roster. Eligible participants were adults aged ≥ 18 years, with type 2 diabetes mellitus that were registered on Primary Care Health Units. who could fill out questionnaires and were mentally stable. There were no exclusion criteria. The research team conducted patient evaluations during home visits, and Community Health Workers accompanied the team to provide additional support.

### Sample size

The SAPPA sampling design is based on the Brazilian Continuous National Household Sample Survey. The target audience includes patients with T2DM registered in Primary Care Health Units in each city. The estimated number of T2DM patients considered the prevalence of diabetes of 5.2% in Amazonas State, Brazil [19]. This sample size was calculated for each city and allowed a confidence level of 99% and a margin of error of 10%. As a result, the analysis included a total of 789 patients. Detailed information on the analysis of missing values is presented in Supplementary Material, Table A1.

### Instruments

**Fig. 2:**
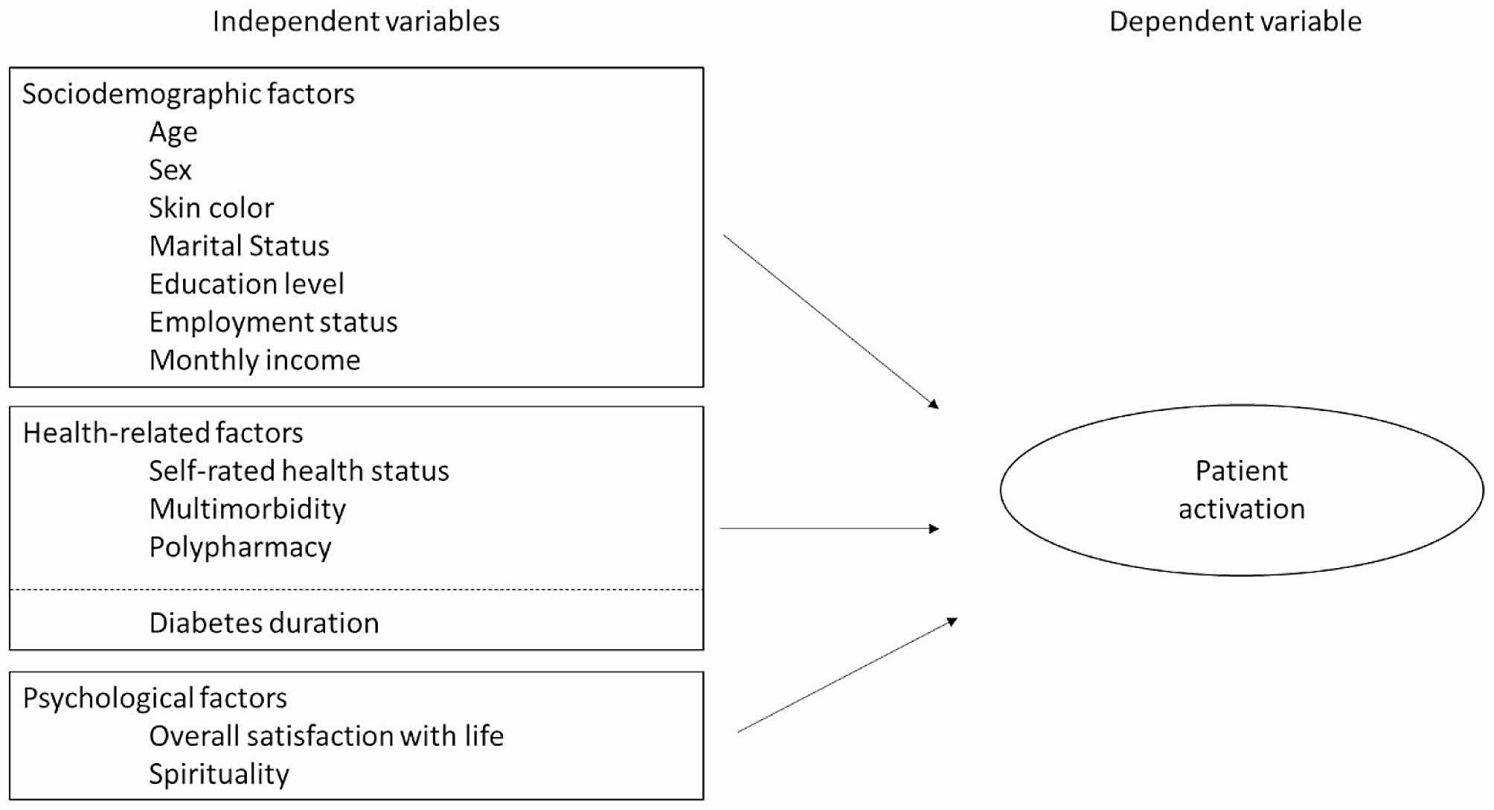
The theoretical model of mechanisms through which different factors might affect diabetic patient activation highlight the importance of individual characteristics

The theoretical framework of this study is shown in Fig. 2). In this study, the dependent variable was Patient activation. The Patient Activation Measure– 13 (PAM-13) is a 13-item measure that assesses self-efficacy, behavior, and knowledge [20]. Individual item scores range from 0 to 4, with 0 “not applicable”; 1 “strongly disagree”; 2 “disagree”; 3 “agree” and 4 “strongly agree”. Mean PAM-13 scores are transformed into a total score ranging from 0 to 100, where higher scores represent higher activation. PAM-13 scores were assigned to one of the four levels of activation, based on the Insignia Health guidelines. PAM Level 1 refers to very low activation (0–47 points) meaning disengaged and overwhelmed; Level 2 (47.1–55.1), becoming aware, but still struggling; Level 3 (55.2–67.0), taking action, and Level 4, high activation (67–100), meaning maintaining behaviors and pushing further [9]. This measurement was adapted and validated for the Brazilian population [21].

### Sociodemographic variables

The study-specific questionnaire assessed sex (female/male), age (years), self-reported skin color (brown/black; white/yellow/indigenous), marital status (married; unmarried), education level (0–4 years; 5 or more), employment status (unemployed; employed), income in minimum wages units (considering the Brazilian minimum wage in Reais received per month, R$ 1,100.00/month is equivalent to USD$ 220.00/month).

### Health-related variables

To collect information on self-rated health perception, participants responded to the question, “In general, compared with other people of our age, you think your health is: worse/much worse or equal or better/much better?”. Multimorbidity was defined as 5 or more self-reported chronic diseases (yes/no) and polypharmacy as the use of 5 or more medications per day (yes/no). Also, patients were asked how long they have been diagnosed with T2DM (diabetes duration, until 5 years; 5–10; 10 years or more).

### Psychological variables

Overall life satisfaction was collected based on the results for the question: “How is your level of satisfaction with your life?” Possible answers were little/very little; enough; very/too much. Intrinsic Religiousness Inventory (IRI) was used to measure spirituality adapted for Brazilian populations [22]. It consists of a structured questionnaire organized on a Likert scale, with scores ranging from 1 to 5 that reflect gradations of intensity/frequency: 1-never, 2-rarely, 3-occasionally, 4-often, and 5-always [23].

### Statistical analysis

All analyses were conducted using IBM SPSS Statistics version 26. In the descriptive analysis, age and diabetes duration were considered continuous variables and reported by mean and standard deviation. Other independent categorical variables were presented by frequency and percentage. One-way ANOVA calculated continuous variables and Chi-square for categorical variables. Regression analysis were only performed with variables with a significant *p*-value (< 0.5). Multicollinearity was performed using the variance inflation factor (VIF) where variables with a VIF > 10 were removed. To assess the association between PAM-13 scores and sociodemographic, health, and psychological factors was applied ordinal logistic regression modeling. Multivariable generalized ordinal logistic regression was used to compare the association of sociodemographic, health, and psychological factors with different PAM-13 levels. Two-tailed *p*-values of less than 0.05 were considered statistically significant.

## Results

Table 1 summarizes the characteristics of the study sample (n = 789 participants). The mean age was 61.7 (standard deviation: 12.9) years. The majority of participants were female (70%; *p* = 0.001), married (55%, *p* = 0.17), self-reported brown or black skin color (84%; *p* = 0.31), low educational level (64%, *p* < 0.001), and low monthly income (65%, *p* < 0.001). Approximately 50% of participants reported to receive less than 1 salary/month. We found no differences in polypharmacy, multimorbidity, and diabetes time diagnosis between PAM levels. There were significant differences in terms of education level, low monthly income, self-rated health perception, and lower overall life satisfaction between PAM levels.

**Table 1:** Descriptive statistics

Variables	Level 1 ≤ 47 (n = 261)	Level 2 (47.1–55.1) (n = 217)	Level 3 (55.2–67.0) (n = 240)	Level 4 (≥ 67.1) (n = 71)	Total (n = 789)	*p*-value
Sex, *n (%)*						
Male	102 (39.1)	73 (33.6)	54 (22.5)	23 (32.4)	252 (31.9)	**0.001** ^*^
Female	159 (60.9)	144 (66.4)	186 (77.5)	48 (67.6)	537 (68.1)	
Age, *mean (sd)*	64.5 (3.3)	62.4 (12.5)	59.1 (12.1)	58.3 (13.1)	61.7 (12.9)	**< 0.001** ^*^
Skin color, n (%)						
Brown/Black	212 (81.2)	184 (84.8)	202 (84.1)	64 (90.1)	662 (83.9)	0.31
White/Yellow/Indigenous	49 (18.8)	33 (15.2)	38 (15.8)	7 (9.9)	127 (16.1)	
Marital status, n (%)						
Married	140 (53.6)	108 (49.8)	144 (60.0)	39 (54.9)	431 (54.6)	0.17
Unmarried	121 (46.4)	109 (50.2)	96 (40.0)	32 (45.1)	358 (45.4)	
Educational attainment (years), n (%)						
0–4	197 (75.5)	143 (52.8)	133 (55.4)	29 (40.8)	502 (63.6)	**< 0.001** ^*^
5+	64 (24.5)	74 (34.2)	107 (44.6)	42 (59.2)	287 (36.4)	
Employment status, n (%)						
Unemployed	176 (67.5)	142 (65.4)	154 (64.2)	36 (50.7)	508 (64.4)	0.07
Employed	85 (35.5)	75 (34.6)	86 (35.8)	35 (49.3)	281 (35.6)	
Income (minimum wage^a^) n (%)						
≤ 1	117 (53.4)	105 (52.8)	96 (43.8)	25 (38.3)	343 (48.7)	**0.01** ^*^
≤ 2	71 (32.4)	54 (27.1)	84 (38.3)	23 (34.3)	232 (33.0)	
> 2	31 (14.1)	40 (20.1)	39 (17.9)	19 (27.4)	129 (18.3)	
Multimorbidity, n (%)						
No	175 (67.0)	128 (59.0)	157 (64.4)	40 (56.3)	500 (63.4)	0.15
Yes	86 (33.0)	89 (41.0)	83 (34.6)	31 (43.7)	289 (36.6)	
Polypharmacy, n (%)						
No	206 (78.9)	177 (81.6)	201 (83.7)	55 (77.5)	639 (81.0)	0.47
Yes	55 (21.1)	40 (18.4)	39 (16.3)	16 (22.5)	150 (19.0)	
Diabetes duration, mean (sd)						
	10.7 (9.3)	10.1 (8.7)	9.8 (7.7)	8.0 (7.2)	10.7 (9.3)	0.17
Self-rated health perception, n (%)						
Worse/much worse	85 (33.6)	54 (25.5)	55 (23.1)	7 (10.0)	201 (26.0)	**< 0.001** ^*^
Equal	84 (33.2)	78 (36.8)	80 (33.6)	17 (24.3)	259 (33.5)	
Better/much better	84 (33.2)	80 (37.7)	103 (43.3)	46 (65.7)	313 (40.5)	
Overall life satisfaction, n (%)						
Little/very little	32 (13.4)	14 (6.5)	20 (8.3)	3 (4.2)	69 (8.8)	**0.016** ^*^
Enough	76 (29.3)	48 (22.2)	51 (21.3)	9 (12.7)	184 (23.4)	
Very/very much	151 (57.3)	154 (71.3)	169 (70.4)	59 (83.1)	533 (67.8)	
Spirituality, *mean (sd)*	47.8 (9.4)	48.2 (5.5)	48.5 (4.7)	48.9 (2.7)	49.2 (8.3)	0.26

^&^ Missing values were presented in Supplementary material Table A1

^a^ Minimum wage = R$ 1,100.00/month or US $ 220.00/month

^*P<0.05. Chi−square test^

Table 2 presents the linear regression analysis results of the association between demographic, clinical, psychosocial factors, and patient activation score. Older age and males were associated with lower PAM-13 scores. Overall, there was a negative relation between patient activation, educational level, and monthly income. Participants with lower overall life satisfaction and worse self-rated health perception presented lower PAM-13 scores.

**Table 2:** Results from linear regression

Variables	Coefficient	*p*-value
Sex (ref = female)		
Male	-2.92	**0.02**
Age	-1.76	**< 0.001**
Education attainment, years (ref = 5+)		
0–4	-4.22	**< 0.001**
Income (minimum wage; ref= > 2)		
≤ 1	-2.535	**0.04**
≤ 2	-1.082	0.40
Self-rated health perception (ref = better/ much better)		
Worse/much worse	-6.271	**< 0.001**
Equal	-3.596	**0.001**
Overall life satisfaction (ref = very/very much)		
Little/very little	-2.296	0.17
Enough	-3.285	**0.03**

R squared = 0.164 (Adjusted R squared = 0.152)

Results of ordinal logistic regression analyses indicated a statistically significant association between sex, age, educational level, self-rated health perception, and overall life satisfaction (Table 3). Compared to women, men were 43% more likely to score lower levels (*p* < 0.001). Results also indicated that older age presented lower PAM-13 levels (*p* < 0.001). Participants with low education levels presented 44% more chance to present lower PAM-13 levels (*p* = 0.03). Worse self-rated health perception (*p* < 0.001), and less overall life satisfaction (*p* = 0.014) were both associated with lower PAM-13 levels.

**Table 3:** Results from ordered logistic regression model

Variables	Exp (B)	Confidence interval	*p*-value
Sex (ref = female)			
Male	0.572	0.429–0.762	< 0.001
Age	0.974	0.963–0.985	< 0.001
Education attainment, years (ref = 5+)			
0–4	0.567	0.457–0.851	0.03
Self-rated health perception (ref = better/ much better)			
Worse/much worse	0.447	0.317–0.632	< 0.001
Equal	0.623	0.457–0.851	0.003
Overall life satisfaction (ref = very/very much)			
Little/very little	0.656	0.483–1.084	0.094
Enough	0.667	0.483–0.920	0.014

## Discussion

Our study examined the link between sociodemographic, health-related factors, psychological factors, and patient activation in a type 2 diabetes mellitus population from Amazonas, Brazil. Our findings show that sex, age, educational level, self-rated health perception, and life satisfaction were significantly associated with patient activation.

In the present study, 33% of the patients were at the lowest activation level 1, about 30% were at level 3, and 27.5% were at level 2. Few patients scored at level 4 (9%). Studies from different countries have shown percentages somewhat different with fewer patients demonstrating low activation and more patients showing high PAM-13 levels, such as Finland [24], the Netherlands [8], and the USA [25, 26]. Similar findings with higher PAM-13 scores are seen among Latino populations who live in the USA [25]. In contrast to other results presented in these studies, the SAPPA sample covers a low-income population with low educational levels, suggesting that socioeconomic factors contribute to the PAM-13 results.

Sociodemographic factors seem to highly interfere in patient activation demonstrating the impact of the social determinants on health. Socioeconomic factors are a multidimensional construct that includes educational, economic, and occupational status [27]. Furthermore, socioeconomic and educational gradients influence diabetes knowledge among primary care patients with T2DM [28]. Social determinants of health were significantly associated with diabetes self-care and outcomes. Our findings corroborate previous studies from a variety of settings and populations including older adults [29], vascular diseases [30], and DM [8, 31] suggesting that individuals with higher education levels and higher income have/experience higher activation.

Income and education level are the highest contributors to overall disparities in health literacy [32]. Health literacy is described as an individual’s ability to obtain, navigate, and understand the basic health information necessary to make an appropriate health-related decision [33]. This capacity is critical for optimal medication adherence [34]. Understanding the potential impact of these factors in managing DM should help decision-makers establish better approaches addressed to the low-income population. Also, highlight to health profession workers the importance of a holistic evaluation of patients with chronic diseases such as DM.

Our findings also suggested that patients with a lower health status show significantly lower patient activation, consistent with previous studies [8, 35–37]. The Patient Activation Measurement tends to present a linear relationship with self-rated health [23]. Health status can be defined as the range of manifestation of disease in a given patient including symptoms, functional limitation, and quality of life, in which quality of life is the discrepancy between actual and desired function [38, 39]. Self-rated health is the factor that is most strongly related to the functional disability of the elderly in Brazil [38]. Therefore, an assessment of the self-rated health of patients with DM is important because it can help monitor treatment guidelines to avoid serious consequences [40]. Older age and worse health reports tend to be factors that contribute to the decline in patient activation over time [41], as found in the present study.

Some limitations should be considered in this survey. This study presented a representative sample of primary care patients in Amazonas, Brazil, limiting the generalizability of the findings to other cultures and geographic regions. Second, there are no anthropometric or biological measurements included in the analysis of this manuscript.

### Practical implications

To further study the impact of PAM on disease management and health outcomes, as well as to understand and take advantage of this new knowledge in practice, we need to explore the relationship between patient activation with DM and relevant patient-reported outcomes in a longitudinal setting. The data from this manuscript motivated an interventional study addressed to community health workers in primary health settings. The first aim of this new study is to develop and test an intervention focused on T2DM management offered by these health professionals. We will measure the impact of this intervention on patient motivation and behavior changes.

Since self-rated health perception and socioeconomic factors were associated with patient activation, healthcare providers could explore these factors when aiming to increase patient activation. This finding supports the need for holistic patient-centered diabetes care in which person-related factors and patient preferences should be taken into account, in addition to disease-related factors.

## Conclusion

Low patient activation was associated with worse sociodemographic, health, and psychological conditions in the Amazon population. The low level of patient activation observed in this sample highlights an important impediment to diabetes disease management/self-management in disadvantaged populations.

Designing interventions that assist patients with chronic diseases to develop skills in practice self-management and shared decision-making is a logical next step in countering health inequalities. Such interventions must be appropriately targeted to the initial level of activation and be culturally sensitive. The Patient Activation Measure appears to provide a viable tool for assessing levels of activation. Next comes the hard work of building effective interventions that support and empower patients [25].

### Electronic supplementary material

Below is the link to the electronic supplementary material.

Supplementary Material 1

Supplementary Material 2

Supplementary Material 3

## Data Availability

The authors confirm that the data supporting the findings of this study are available within the supplementary materials.

## Abbreviations

T2DM: Diabetes Mellitus Type 2
DM: Diabetes Mellitus
SAPPA: Study of Health in Primary Care of the Amazon Population
PAM-13: Patient Activation Measure-13
IRI: Intrinsic Religiousness Inventory
VIF: Variance inflation factor
