# Supplementary material for: Patient activation levels and socioeconomic factors among the Amazonas population with diabetes: a cross-sectional study
Source: BMC Health Serv Res. 2024 Feb 6;24:169. doi: 10.1186/s12913-023-10529-0 (PMC10848446; doi:10.1186/s12913-023-10529-0)
Supplement: Supplementary file 1 — Supplementary Material 1 [file 12913_2023_10529_MOESM1_ESM.docx]

Supplementary material

Table A1 Number and proportion of missing values among all study variables.

| Variables | Total  n=789 | Missing (%) |
| --- | --- | --- |
| Sex | 789 | 0 |
| Age | 768 | 21 |
| Skin color | 789 | 0 |
| Marital status | 789 | 0 |
| Educational level (years) | 789 | 0 |
| Employment status | 789 | 0 |
| Income | 704 | 85 |
| Self-rated health perception | 773 | 16 |
| Multimorbidity (≥5 chronic diseases) | 789 | 0 |
| Polypharmacy | 789 | 0 |
| Diabetes duration since diagnosis (years) | 787 | 2 |
| Overall life satisfaction | 786 | 3 |
| Spirituality | 756 | 33 |

Table A2

| Variables | PAM score | Total | *p*-value |
| --- | --- | --- | --- |
| Sex (n=789) |  |  |  |
| Female | 53.42 ± 11.91 | 537 | 0.004 |
| Male | 50.72 ± 12.82 | 252 |  |
| Age (n=772) |  |  |  |
| 20-49 | 55.57 ± 12.42 | 140 | <0.0001 |
| 50-59 | 54.50 ± 13.58 | 180 |  |
| 60-69 | 52.06 ± 12.08 | 230 |  |
| 70+ | 48.69 ± 12.27 | 222 |  |
| Skin color (n=789) |  |  |  |
| Brown | 53.86 ± 12.37 | 640 | 0.63 |
| Black | 52.93 ± 12.41 | 22 |  |
| White | 52.23 ± 10.99 | 106 |  |
| Yellow | 54.08 ± 14.58 | 8 |  |
| Indigenous | 56.02 ± 11.39 | 13 |  |
| Marital status (n=789) |  |  |  |
| Married | 52.54 ± 12.48 | 431 | 0.79 |
| Unmarried | 52.26 ± 17.47 | 358 |  |
| Educational level (years) |  |  |  |
| 0-4 | 49.14 ± 11.81 | 502 | 0.0001 |
| 5-8 | 51.71 ± 12.86 | 54 |  |
| 9+ | 57.71 ± 13.22 | 233 |  |
| Employment status (n=789) |  |  |  |
| Unemployed | 51.03 ± 12.60 | 508 | 0.003 |
| Employed | 53.92 ± 12.90 | 281 |  |
| Income (n=704) | |  |  |
| 0-1 | 50.99 ± 12.45 | 343 | 0.009 |
| l-2 | 53.37 ± 12.83 | 232 |  |
| 2-3 | 54.08 ± 12.64 | 67 |  |
| 3+ | 56.00 ± 14.13 | 62 |  |
| Self-rated health perception (n=773) |  |  | <0.0001 |
| Worse/ much worse | 48.79 ± 11.75 | 201 |  |
| Equal | 51.69 ± 12.03 | 259 |  |
| Better/ much better | 54.94 ± 13.26 | 313 |  |
| Multimorbidity (≥5 chronic diseases) |  |  |  |
| No | 51.68 ± 13.02 | 500 | 0.22 |
| Yes | 52.83 ± 12.32 | 289 |  |
| Polypharmacy (n=789) |  |  |  |
| No | 52.18 ± 12.84 | 639 | 0.69 |
| Yes | 51.72 ± 12.54 | 150 |  |
| Diabetes duration since diagnosis (years) |  |  |  |
| <5 | 52.59 ± 14.55 | 240 | 0.08 |
| 5-10 | 53.41 ± 11.78 | 199 |  |
| >10 | 51.00 ± 11.96 | 348 |  |
| Overall life satisfaction (n=786) |  |  |  |
| Little/very little | 48.83 ± 12.88 | 69 | <0.0001 |
| Neutral | 49.45 ± 12.08 | 184 |  |
| Very/very much | 53.52 ± 12.76 | 533 |  |

Table A3

| Variables | VIF |
| --- | --- |
| Sex | 1.065 |
| Age | 1.228 |
| Skin color | 1.016 |
| Marital status | 1.011 |
| Educational level (years) | 1.201 |
| Employment status | 1.164 |
| Income | 1.084 |
| Self-rated health perception | 1.137 |
| Multimorbidity (≥5 chronic diseases) | 1.044 |
| Polypharmacy | 1.029 |
| Diabetes duration since diagnosis (years) | 1.058 |
| Overall life satisfaction | 1.111 |
