# Supplementary material for: Patient activation levels and socioeconomic factors among the Amazonas population with diabetes: a cross-sectional study
Source: BMC Health Serv Res. 2024 Feb 6;24:169. doi: 10.1186/s12913-023-10529-0 (PMC10848446; doi:10.1186/s12913-023-10529-0)
Supplement: Supplementary file 3 — Supplementary Material 3 [file 12913_2023_10529_MOESM3_ESM.pdf]

EMBODIED OPINION OF THE CEP

SPLICE DATA

Research Title: SAPPA STUDY (Study of Health in Primary Care of the Amazonian Population) Researcher: Elisa Brosina de Leon
Thematic Area:
Version: 5
CAAE: 25030719.4.0000.5020
Proposing Institution: Federal University of Amazonas - UFAM
Lead Sponsor: Own Funding

DETAILS OF THE OPINION

Opinion Number: 4.318.325

Project Presentation:
Changes in the light of CONEP's recommendation for the Embodied Opinion

PROJECT PRESENTATION
This is a relevant study, a Research Project of the Universal Call for Proposals - Foundation for Research Support in the State of Amazonas (FAPEAM) of 2018, which has Profa. Dr. Elisa Brosina from the Bachelor's Degree in Physical Therapy at the Faculty of Physical Education and Physical Therapy of the Federal University of Amazonas, which involves several co-participating centers, duly registered on the Brazil platform. The research was maintained with the same objectives and methodological procedures, as approved by this committee. However, as it is an amendment presented here, it has undergone a small increase without disfiguring the original project.

According to the researcher: This research aims to support health services aimed at the care of people with chronic conditions. The objective of this study was to evaluate the institutional capacity for care for chronic conditions in health services in the interior of the State of Amazonas. Using the Assessment of Chronic IIlness Care (ACIC) and Patient Assessment of Chronic IIlness Care (PACIC) instruments as a method. The physical-functional evaluation of UBS users will also be included. It is hoped that this research will describe the ability to assistance from health services in the interior of Amazonas, according to the criteria of organization of the health system; community resources; support for self-care; design of the care line; clinical decision support; The objective of this study was to identify the most relevant points to be rethought in order to increase the quality of comprehensive care provided to people with chronic conditions, as well as to describe the repercussions on the functional capacity of the users served.

Proposed Methodology:

Study Participants: 1560 (health professionals = 390 and UBS users = 1,170)

Study Design: This is an exploratory, cross-sectional study based on data that will be obtained through the application of ACIC and PACIC. Period and location of the Study: Data collection will be carried out from January 2020 to June 2021 in the Basic Health Units (UBS) in the cities of Careio da Várzea, Iranduba, Itacoatiara, Manacapuru, Novo Airão, Presidente Figueiredo, Rio Preto da Eva, Manaquiri, Autazes, Careiro Castanho, Silves, Itapiranga and Manaus, after approval from the Research Ethics Committee (CEP) involving human beings and consent from the health departments of the municipalities. Study Population: The metropolitan region (MR) of Manaus is composed of twelve municipalities, namely Careio da Várzea, Iranduba, Itacoatiara, Manacapuru, Novo Ayrão, Presidente Figueiredo, Rio Preto da Eva, Maniquiri, Autazes, Careiro Castanho, Silves and Itapiranga, in addition to Manaus. Moment 1: 3 UBS), located in the urban areas of each city in the Metropolitan Region of Manaus, will be randomly drawn. All units will have an equal chance of being drawn. All health professionals belonging to the units will be invited to participate in the study. First, the study will be presented to the municipal health secretaries and to the managers of the basic health units for appreciation of the objectives and main results to be achieved with the application of the proposed instruments. Subsequently, the professionals of the units will be invited to fill out the questionnaire of Evaluation of Institutional Capacity for Care of Chronic Conditions (ACIC) by electronic means. The e-mail addresses will be requested from the Municipal Health Departments of each of the municipalities involved. It is expected to approach 10 professionals in each unit, totaling 390 professionals. Moment 2: The evaluation of the quality of care provided to patients with chronic diseases will be evaluated in loco through the application of a questionnaire with users of the evaluated UBSs. On a date agreed with the municipal health secretary and the management of the UBS, users will be invited to participate in a meeting in which the project will be presented and later the PACIC questionnaire will be applied by the health team. Researchers. People diagnosed with a chronic disease such as diabetes mellitus, hypertension or both self-reported or confirmed by the medical record, followed by the UBS for at least one year, will be included. Patients with severe cognitive impairment (score on the Mini Mental State Examination - MMSE 9) will be excluded, in the absence of a responsible person who could assist in the answers. For the sample calculation, the method proposed by Lwanga and Lemeshow (NAING, 2017) will be used in order to verify whether the n of the present study is sufficient to assess the quality of care provided by the primary level of health care. To this end, we considered the mean prevalence of good quality of care in PHC of 39.7% (ARAÚJO et al, 2014), absolute precision of 10% and significance level of 5%, considering an estimate of 250 patients treated annually at the UBS (MOTA, 2014). A sample size of 81 individuals is estimated in each city evaluated. Considering 10% of possible losses, the estimated total n was 90 individuals. Therefore, the user population evaluated in each UBS will consist of 30 individuals.

Data Analysis Methodology:

Data will be analyzed using the Statistical Package for Social Sciences (SPSS, version 23.0, Chicago, IL, USA). First, the results will be analyzed using descriptive techniques, expressed in proportions or

percentages for categorical and median variables with their respective interquartile ranges (IQR) for non-parametric continuous variables. The reliability analysis of the ACIC and PACIC questionnaires will be performed using Cronbach's alpha coefficient. Spearman's-Rho correlation test of ACIC scores and total PACIC will be performed between the dimensions of the questionnaire. Comparison of the median of the total ACIC and PACIC scores with the care outcomes (body mass index, blood pressure levels, and the two WHOQOL-bref questions used) will be performed using the Mann-Whitney U test and subsequently adjusted for potential confounding variables (gender, age, education, income, time since diagnosis of diabetes mellitus, and time since diagnosis of hypertension) in the multivariate model using backward logistic regression. The level of significance adopted will be 5%. The values obtained will be expressed as odds ratios (OR) and their respective 95% confidence intervals (95% CI) (SILVA et al, 2018).

INCLUSION AND EXCLUSION CRITERIA

Inclusion

Moment 1: Health professionals who provide service at the UBS for at least 3 months from the date of invitation to participate in the study with or without remuneration.

Moment 2: Patients diagnosed with a chronic disease such as diabetes mellitus, hypertension or both self-reported or confirmed by the medical records, followed by the UBS for at least one year.

Exclusion

Health professionals who do not provide direct care to the patient will be excluded;

Professionals who are on leave for vacation or vacation;

Patients with severe cognitive impairment (score on the Mini Mental State Examination - MMSE 9), in the absence of a responsible person who could assist in the answers.

Research Objective:

According to the researcher General

Objective

OBJECTIVE: To evaluate the institutional capacity for care for chronic conditions in health services in the interior of the State of Amazonas.

Specific Objectives

- OBJECTIVE: To evaluate the institutional capacity of primary health care units in the Metropolitan Region of Manaus to provide care for users' chronic conditions according to the Chronic Conditions Care Model.
- To evaluate the quality of care for users with chronic diseases in health units in Primary Health Care according to the Chronic Conditions Care Model from the user's own perspective.
- Support health teams and managers in the development of strategies that strengthen the potential and minimize weaknesses in the care of chronic conditions.
- To correlate the scores obtained in the instruments used with the Municipal Human Development Index.

Assessment of Risks and Benefits:

According to the researcher
Risks:
All research with human subjects involves risks. The risk of conducting the research involves the possibility of psychological embarrassment for health professionals and for patients to answer the questionnaire about the potential weaknesses of the institution where they work or where they are cared for, respectively. In order to minimize the risk, the invitation to participate will be made individually, in a room previously defined for the application of the research. Participants will not be identified by name, and a code will be used to tabulate the data. The final results will be presented using an average so that it is not possible to identify the research participants, neither by the general public nor by the health administrator. All the questions in the questionnaire are directed to the functioning of the UBS and do not aim to obtain answers about the behavior of the professionals or the care provided by them. If there is a need to travel to participate in the research, the research team will be responsible for the transportation and food costs related to the applications of the instruments.

Benefits:
The knowledge of the weaknesses presented by the institution in the care of chronic diseases will allow the development of strategies aimed at strengthening the care model provided to patients with chronic conditions.

Comments and Considerations on the Research:
This is an amendment to the Research Project of the Universal Call for Proposals of FAPEAM 2018, which has Profa. Dr. Elisa Brosina from the Bachelor's Degree in Physical Therapy at the Faculty of Physical Education and Physical Therapy of the Federal University of Amazonas, and team members Mérida Zilanda Barbosa Bordoni, Elorides de Brito, MAIRA MENDES DOS SANTOS MARQUES and EULER ESTEVES RIBEIRO.

Mandatory Submission Terms Considerations:

1. Cover Page: PROPER - PB_INFORMAÇÕES_BÁSICAS_1625966_E1.pdf 05/11/2019, 08:27:26
2. Terms of Consent: ADEQUATE, AS IT WAS ATTACHED TO THE PROTOCOL;
3. Research Instruments: ADEQUATE, AS IT WAS ATTACHED TO THE PROTOCOL;
4. Research Project: ADEQUATE;
5. PROFESSIONAL INFORMED CONSENT FORM: ADEQUATE;

6. USER INFORMED CONSENT FORM: ADEQUATE;
7. Curriculum Lattes: ADEQUATE, AS IT WAS ATTACHED TO THE PROTOCOL.

Recommendations:

See field to-do list and inadequacies

it is recommended to observe the guidelines of CONEP, of 5/6/2020.

it is recommended to observe the guidelines of PROPESP/UFAM, through official letter 009/2020, which establishes procedures for research activities with face-to-face human beings. See pp. 2/5 and 3/5, which mentions that face-to-face research with human subjects should be suspended during the pandemic.

Conclusions or Pending Issues and List of Inadequacies:

No ethical obstacles were found

In view of the above, we are of the opinion that the Amendment to the project be APPROVED, as the researcher complied with the determinations of Res. 466/2012.

That is the opinion

Final Considerations at the discretion of the CEP:

This opinion was prepared on the basis of the **documents listed below:**

Document Type	Archive	Post	Author	Situation
Basic Information of the Project	PB_INFORMAÇÕES BÁSICAS_1625966 E1.pdf	22/09/2020 17:36:24		Accepted
Detailed Design / Researcher Brochure	Zip Code Project.pdf	22/09/2020 17:35:23	Elisa Brosina de Leon	Accepted
Declaration of Institution and Infrastructure	Sudam consent.pdf	05/03/2020 11:05:22	Elisa Brosina de Leon	Accepted
Other	Zip Code.pdf	02/12/2019 15:34:56	Elisa Brosina de Leon	Accepted
Other	TCLEusersNEW.pdf	02/12/2019 15:34:09	Elisa Brosina de Leon	Acepeted

ICF / Terms of Assent / Justification of Absence	ICFprofessionalsNEW.pdf	02/12/2019 15:31:15	Elisa Brosina de Leon	Accepted
Cover Page	Folhaface.pdf	05/11/2019 08:27:26	Elisa Brosina de Leon	Accepted
Declaration of the Sponsor Decision	Universal.pdf	04/11/2019 11:50:47	Elisa Brosina de Leon	Accept
Other	1_Elisa_Brosina.pdf	29/10/2019 18:44:34	Merida Zilanda Barbosa Bordoni	Accepted

Status of the Opinion:
Approved

CONEP needs to be assessed:
No

MANAUS, October 04, 2020

Signed by:
Eliana Maria Pereira da Fonseca
(Coordinator)

Address: Rua Teresina, 495
Neighborhood: Adrianópolis **Zip Code: 69.057-070**
UF: AM **Municipality:** MANAUS
Phone: (92)3305-1181 **Email:** cep.ufam@gmail.com
